# Stratifying primary Sjögren’s syndrome: killers in the balance?

**DOI:** 10.1186/s13075-015-0878-9

**Published:** 2015-12-07

**Authors:** Simon J. Bowman, Benjamin A. Fisher

**Affiliations:** Rheumatology Research Group, University of Birmingham, Edgbaston, Birmingham, B15 2WD UK; Department of Rheumatology, University Hospitals Birmingham NHS Trust, Queen Elizabeth Hospital Birmingham, Mindelsohn Way, Edgbaston, Birmingham, B15 2TH UK

## Abstract

The article by Seror et al. in *Arthritis Research & Therapy* reports data from the 15 French patients in the open-label BELISS (Efficacy and Safety of Belimumab in Subjects With Primary Sjögren's Syndrome, NCT01160666) study of belimumab in primary Sjögren’s syndrome. The study identifies that higher baseline levels of natural killer cells in the peripheral blood and salivary glands are associated with non-response to belimumab therapy. Although caution is required given the open-label nature of the trial, this study adds to data already suggesting a role for natural killer cells in primary Sjögren’s syndrome and, importantly, indicates a need for therapeutic stratification.

Primary Sjögren’s syndrome (pSS) is characterised by lymphocytic infiltration of the salivary and lacrimal glands leading to symptoms of dryness and systemic manifestations in 30–40 % of patients. B-lymphocyte hyperactivity is considered a key feature of pathogenesis and results in B-cell lymphomas in 5 % of patients. Levels of the B-cell activating factor (BAFF) (also known as B-lymphocyte stimulator) are associated with markers of B-cell hyperactivity in pSS, and a mouse model overexpressing BAFF has features of pSS and increased lymphoma risk. BAFF therefore offers a promising therapeutic target.

The article by Seror et al. [1] , in *Arthritis Research & Therapy* makes an important observation relevant to treatment and pathogenesis of pSS, based on an analysis of samples from the BELISS (Efficacy and Safety of Belimumab in Subjects With Primary Sjögren's Syndrome, NCT01160666) study. BELISS included 15 patients from France and 15 from Italy. Patients received belimumab, a monoclonal antibody against BAFF, at 0, 2 and 4 weeks and then every 4 weeks for up to a year. Clinical data have already been published [2], but in the French cohort studied here, an improvement in the EULAR (European League Against Rheumatism) Sjögren’s Syndrome Disease Activity Index (ESSDAI) was observed in 6 out of the 15 patients.

We strongly welcome the biologic era in pSS. Although pSS is not associated with increased mortality (except for the small number of patients with aggressive forms of lymphoma), it is associated with significant and disabling symptoms and little evidence that conventional immunosuppressant therapy is effective. In terms of the clinical data from BELISS, there is an obligatory note of caution: a fully powered double-blind randomized controlled trial (RCT) of infliximab in pSS failed to demonstrate improvement [3], despite encouraging data from a pilot open-label study such as this one. Furthermore, despite promising data from two small RCTs, a French phase IIb study of the anti-CD20 monoclonal antibody rituximab, targeting B cells, did not meet its primary outcome, despite significant beneficial effects on fatigue [4]. The reasons for this latter discrepancy are unclear but include methodological issues related to patient selection and outcome measures as well as those relating to target selection such as co-depletion of regulatory B cells and potential protection of pathogenic B cells in tissue niches. Some of these issues may be clarified by a UK study due to report later this year (http://public.ukcrn.org.uk/search/StudyDetail.aspx?StudyID=9809). However, alongside B cells, there is evidence of an important pathogenic role for T cells, epithelial cells, natural killer (NK) cells, and type I and II interferons (IFNα/β and IFNγ, respectively). Analysis of minor salivary glands (MSGs) from an earlier open-label study of rituximab pointed to baseline differences in gene expression between responders and non-responders relating to B-cell and IFN pathways [5]. This suggests the tantalising possibility that patients may be stratified according to dominant pathogenic processes, and it is in regard to this that the study by Seror et al. is of greatest interest.

The authors studied peripheral blood lymphocyte subsets and MSGs at weeks 0 and 28. The only findings associated with systemic response to belimumab were a lower percentage and number of peripheral NK cells and a smaller NK cell infiltrate around lymphocytic foci in the MSGs. Furthermore, the blood NK cell number rose in non-responders but not in responders at week 28. A role for NK cells in pSS is supported by a genetic susceptibility association with the *NCR3* locus, encoding the NK-specific activating receptor NKp30 [6]. This polymorphism is associated with greater IFNγ expression by NK cells. NK cells also enhance production of dendritic cell interleukin-12 (IL-12), which is a key differentiation factor for T helper 1 (Th1) cells, themselves a major source of IFNγ [7]. The data reported by Seror et al. might support the proposal of two dominant pathogenic axes, type I IFN/B cells and IFNγ/IL-12. However, it should be emphasised that there is crosstalk between these two axes and they are not discrete. IFNγ as well as type I IFN can drive BAFF expression [7], and B cells can produce IFNγ. Recent data have also suggested that a high MSG IFNγ, rather than IFNα, signature is associated with lymphoma development [8]. Both NK cells and IFNγ have been associated with the focus score [6, 9], so it is also possible that NK cell infiltration may simply be associated with severity. Although the authors report a fall in the lymphocytic focus score (preferred over the Chisholm and Mason criteria [10]), this did not reach statistical significance, and they did not report whether baseline focus score correlated with NK infiltration or whether change in focus score was associated with response. This may require a larger sample size or a theoretically more sensitive measure such as focus score area [10]. Furthermore, it is not clear from the limited published data available how well NK cell number would function as a predictive biomarker in the clinic. Nevertheless, these exciting findings point to a need for stratification in pSS and hint at an exciting future of personalised medicine in pSS.

## Abbreviations

BAFF: B-cell activating factor
BELISS: Efficacy and Safety of Belimumab in Subjects With Primary Sjögren's Syndrome
CD: Cluster of differentiation
IFN: Interferon
IL: Interleukin
MSG: Minor salivary gland
NK: Natural killer
pSS: Primary Sjögren’s syndrome
RCT: Randomized controlled trial
